# TRPC6 Inactivation Reduces Albuminuria Induced by Protein Overload in Sprague Dawley Rats

**DOI:** 10.3390/cells11131985

**Published:** 2022-06-21

**Authors:** Eun Young Kim, Stuart E. Dryer

**Affiliations:** 1 Department of Biology and Biochemistry, University of Houston, Houston, TX 77204, USA; ekim8@central.uh.edu; 2 Department of Biomedical Sciences, Tilman J. Fertitta Family College of Medicine, University of Houston, Houston, TX 77204, USA

**Keywords:** TRPC6, TRPC5, glomerular physiology, tubulointerstitial fibrosis, albuminuria

## Abstract

Canonical transient receptor potential-6 (*TRPC6*) channels have been implicated in familial and acquired forms of focal and segmental glomerulosclerosis (FSGS), and in renal fibrosis following ureteral obstruction in mice. TRPC6 channels also appear to play a role in driving glomerular disease in aging and in autoimmune glomerulonephritis. In the present study, we examine the role of TRPC6 in the proteinuric state caused by prolonged albumin overload (AO) in Sprague Dawley rats induced by daily injections of exogenous albumin. This was assessed in rats with a global and constitutive inactivation of TRPC6 channels (*Trpc6*^del/del^ rats) and in wild-type littermates (*Trpc6*^wt/wt^ rats). AO for 14 and 28 days caused increased urine albumin excretion that was significantly attenuated in *Trpc6*^del/del^ rats compared to *Trpc6*^wt/wt^ controls. AO overload did not induce significant glomerulosclerosis or azotemia in either genotype. AO induced mild tubulointerstitial disease characterized by fibrosis, hypercellularity and increased expression of markers of fibrosis and inflammation. Those changes were equally severe in *Trpc6*^wt/wt^ and *Trpc6*^del/del^ rats. Immunoblot analysis of renal cortex indicated that AO increased the abundances of TRPC3 and TRPC6, and caused a nearly complete loss of TRPC5 in *Trpc6*^wt/wt^ rats. The increase in TRPC3 and the loss of TRPC5 occurred to the same extent in *Trpc6*^del/del^ rats. These data also suggest that TRPC6 plays a role in the normal function of the glomerular filtration barrier. However, whether TRPC6 inactivation protects the tubulointerstitial compartments in Sprague Dawley rats depends on the disease model examined.

## 1. Introduction

Canonical transient receptor potential-6 (TRPC6) channels are Ca^2+^-permeable cationic channels expressed in many cell types, including podocytes and mesangial cells within renal glomeruli [1]. In podocytes these channels mediate Ca^2+^ signaling in response to signals mediated by circulating and endocrine factors, such as ATP and angiotensin II [2,3,4], and they can also become active in response to mechanical stimuli both in vitro [5] and in vivo, for example, in response to increases in intraglomerular capillary pressure [6].

In 2005, mutations in TRPC6, the gene that encodes TRPC6 channels, were identified in patients with familial forms of focal and segmental glomerulosclerosis (FSGS) [7,8]. The glomerular disease associated with these mutations occurred with an adult onset, progressed to end-stage kidney disease (ESKD), and had an autosomal dominant mode of transmission. Since those initial reports, several other TRPC6 mutations have been identified in familial forms of FSGS [9,10,11,12,13,14]. The majority of these mutations encode cation channels that exhibit a marked gain of function when they are expressed in heterologous systems (reviewed in [1]). It was subsequently shown that expression of wild-type TRPC6 proteins and transcripts is increased in patients with acquired forms of glomerular disease, including FSGS and minimal change disease [15]. Moreover, podocyte-specific over-expression of wild-type or mutant TRPC6 channels in mice resulted in glomerulosclerosis and albuminuria [16,17] indicating that the dysfunction of TRPC6 channels in podocytes is sufficient to drive kidney disease. These results suggest that TRPC6 might be a useful therapeutic target in at least certain forms of chronic kidney disease, especially proteinuric diseases characterized by an early loss of podocytes. They also suggest that TRPC6 plays an as yet poorly defined in regulating the normal function of the glomerular filtration barrier.

If TRPC6 channels are therapeutically useful targets, then the inhibition or inactivation of TRPC6 channels should reduce various indices of kidney disease in animal models in which glomerular function is altered. This has been studied in certain disease models. For example, in the chronic puromycin aminonucleoside (PAN) model of adaptive FSGS in Sprague Dawley rats, there are marked increases in glomerular TRPC6 abundance [18] that result in markedly elevated cation currents in podocytes in situ when these currents are evoked by membrane stretch [1]. This is also observed in cultured podocytes exposed to circulating factors implicated in primary FSGS [19,20]. Moreover, in the chronic PAN model, CRISPR/Cas9-mediated inactivation of TRPC6 resulted in robust reductions in albuminuria, uremia, azotemia, glomerulosclerosis, foot process effacement, and tubulointerstitial fibrosis [18]. TRPC6 inactivation also produced a similar reduction in glomerulosclerosis in anti-GBM models of autoimmune glomerulonephritis [21] and in aging [22] in Sprague Dawley rats. However, in those models, the inactivation of TRPC6 did not reduce albuminuria or tubulointerstitial disease, in spite of the reductions in glomerulosclerosis. Similarly, TRPC6 inactivation did not reduce albuminuria in rats with STZ-induced diabetes [23,24], although it may reduce foot process effacement and podocyte detachment on at least some genetic backgrounds [23]. TRPC6 knockout in mice resulted in marked reductions in tubulointerstitial fibrosis observed after unilateral ureteral obstruction [25,26,27]. Collectively, these data support TRPC6 inhibition as a therapeutic strategy for several forms of kidney disease, although the nature and extent of the protective effect may depend on the actual disease model, the species, and the genetic background.

It has long been known that rats develop kidney disease if they are caused to become hyperalbuminemic as a result of chronic administration of exogenous albumin [28,29,30,31]. This is a complex model in which tubulointerstitial disease and proximal tubule injury are the most prominent features [32,33]. However, podocyte involvement has also been suggested. Thus, podocytes take up serum albumin by endocytosis [34], which results in increased p38 MAP kinase and TGFβ signaling, and loss of synaptopodin [35,36], a cytoskeletal protein that is important for maintaining foot process ultrastructure in response to stress [37]. Albumin overload in vitro can trigger marked cytoskeletal rearrangements and apoptosis in podocytes [38,39]. Importantly, exposing cultured podocyte to elevated albumin also induces increases in Ca^2+^ influx that can be blocked by siRNA knockdown of TRPC6 or by pharmacological inhibition of TRP channels [39]. One study has indirectly implicated TRPC6 in disorders of glomerular function caused by AO. Thus, the knockout of Sdc4, the gene that encodes syndecan-4 reduces albumin secretion evoked by albumin overload, and caused an ~80% reduction in TRPC6 protein and transcripts in mice, as well as a marked reduction in Ca^2+^ influx into podocytes [40].

In the present study, we examine the effect of TRPC6 inactivation in a four-week albumin overload model of kidney disease in Sprague Dawley rats. The current study reports that renal cortical TRPC6 expression is markedly increased in this model, whereas glomerular TRPC5 expression is largely suppressed. Moreover, TRPC6 inactivation by CRISPR/Cas9 methods results in substantial reductions in albuminuria in response to albumin overload. Mild histological and biochemical manifestations of kidney disease are found primarily in the tubulointerstitial compartment as a result of albumin overload, and these do not appear to be ameliorated by TRPC6 inactivation.

## 2. Materials and Methods

### 2.1. Animals

All procedures used in animal experiments were approved by the University of Houston Institutional Animal Care and Use Committee, following NIH and ARRIVE guidelines. The Sprague Dawley *Trpc6*^wt/wt^ and *Trpc6*^del/del^ rats used in these experiments were previously described in detail [18]. Briefly, CRISPR/Cas9 methods were used to introduce a 239-bp deletion within Exon 2 of *Trpc6*. As a result of this gene edit, the *Trpc6*^del/del^ rats splice out all of Exon 2 during transcription. Small amounts of the truncated protein were produced, but those channels were not functional, and hence this deletion functioned in the same way as a global and constitutive *Trpc6* knockout. TRPC6 currents were not present in the glomerular cells isolated from *Trpc6*^del/del^ rats, but were readily detected in *Trpc6*^wt/wt^ littermates [18].

### 2.2. Albumin Overload and Histology

**Figure 1:**
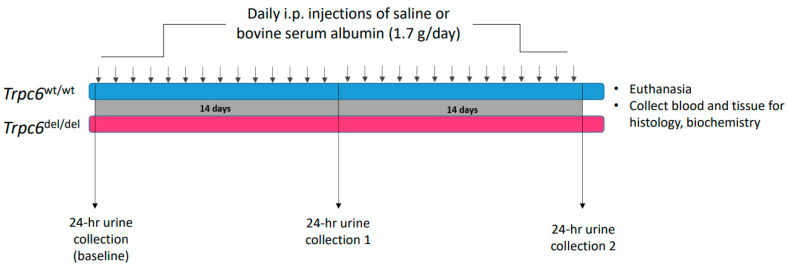
Schematic diagram showing the experimental design used in this study. Bovine serum albumin (BSA) or saline were injected daily over a period of 28 days. The 24 h urine samples were collected just before the protocol, after 14 days, and at 28 days, and used for evaluation of urine albumin excretion. Experiments were performed on wild-type rats (*Trpc6*^wt/wt^) and homozygous littermates that did not have functional TRPC6 channels (*Trpc6*^del/del^).

A schematic diagram of this protocol is shown in Figure 1. The animals were given i.p. injections of bovine serum albumin (BSA) dissolved in sterile normal saline at a dose of 1.7 g/day each day for 28 days. Control animals received equal volumes of saline vehicle. Urine albumin in 24 h urine samples was measured using a commercial ELISA assay (Exocell Inc., Philadelphia, PA, USA) prior to BSA injections, after 14 days of albumin injections and at the end of the 28-day protocol. On the day following the final BSA injection, blood was sampled and the animals were sacrificed by CO_2_ inhalation followed by cervical dislocation, and the kidneys were excised and weighed and used for biochemical and histological analyses. Blood urea nitrogen (BUN) was then determined using a kit from Arbor Assays (San Jose, CA, USA). Portions of kidney from animals in each group were fixed by immersion in 10% buffered formalin, embedded in paraffin, and 4 µm sections were stained using periodic acid-Schiff’s (PAS) or Masson’s trichrome methods, as previously described in detail [18,21,22], and the sections stained by Masson’s method were evaluated using a semi-quantitative scale by an observer blind to genotype or treatment group [22]. Briefly, for each animal, at least 20 tubulointerstitial fields were examined using microscopes with 20× objectives for the presence of tubular dilatation, tubular atrophy, protein casts, interstitial infiltrates, and interstitial fibrosis. Using these criteria, each section was graded as (0) normal; (1) mild change; (2) moderate change; or (3) severe change.

### 2.3. Immunoblot Analysis

These methods were previously described in detail [18]. Rabbit antibodies against TRPC6 (ACC-017) and TRPC3 (ACC-016) were obtained from Alomone Labs (Jerusalem, Israel). A mouse monoclonal antibody against TRPC5 was obtained from NeuroMAB (Davis, CA, USA). All TRPC antibodies were used at a dilution of 1:1000. The antibody against vimentin (cloneV9) was from DAKO (Agilent, Santa Clara, CA, USA) and used at a dilution of 1:1000. The antibody against NOD-, LRR-, and pyrin domain-containing protein 3 (NLRP3) was obtained from LSBio (Seattle, WA, USA), and was used at a dilution of 1:1000.

### 2.4. Statistical Analyses

Statistical analyses were performed using public-access computational tools (http://www.vassarstats.net. Data on urine albumin excretion were analyzed by two-way ANOVA followed by Tukey’s Honest Significant Difference post hoc test. The two independent variables were genotype (*Trpc6*^wt/wt^ vs. *Trpc6*^del/del^) and treatment (daily BSA vs. daily saline). A statistically positive result was inferred when *F* values for the interaction between treatment effects and genotype indicated *p* < 0.05. Bonferroni *t*-test was used in other analyses. All bar graphs in this study show mean ± SD. Densitometric analyses of immunoblot experiments are presented as fold changes relative to the lowest value observed in a control group.

## 3. Results

**Figure 2:**
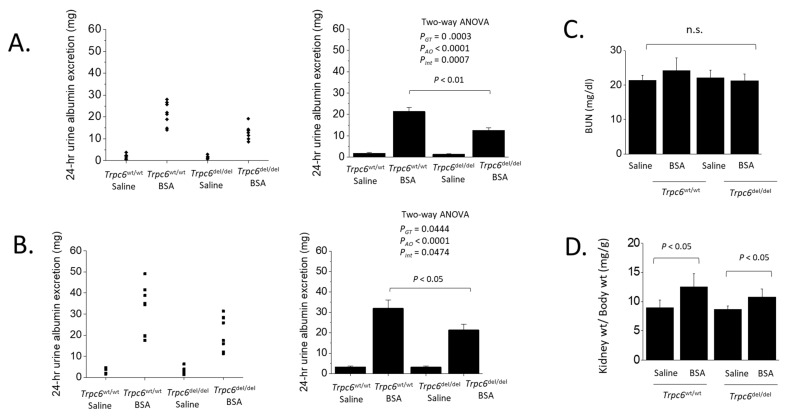
TRPC6 inactivation reduces albuminuria in the albumin overload model. These data were obtained after 14 (**A**) and 28 days (**B**) of daily injections of saline or BSA, as indicated. Each group had eight animals. Data are shown as scatter plots (**left**) and as bar graphs (**right**) indicating mean ± SD. Two-way ANOVA indicates significant effects of BSA treatment, genotype, as well as a significant interaction effect between BSA treatment and genotype indicating a protective effect of TRPC6 inactivation. (**C**) These protocols did not result in increased blood urea nitrogen (BUN) in any group. (**D**) Albumin overload increased kidney weight:body weight ratio in *Trpc6*^wt/wt^ and *Trpc6*^del/del^ rats.

The wild-type (*Trpc6*^wt/wt^) rats and littermates that were homozygous for a deletion in exon 2 of *Trpc6* (*Trpc6*^del/del^) were previously described, and it is important to note that this deletion resulted in TRPC6 channels that were non-functional [18]. The albumin overload model was implemented by daily i.p. injections of normal saline or saline containing bovine serum albumin (BSA) at a daily dose of 1.7 g of BSA. These daily injections were performed over a period of 28 days (Figure 1). Urine albumin was detected at very low levels in all four groups of animals prior to the onset of daily injections, and this was also true for rats that received daily saline injections for four weeks, regardless of genotype (Figure 2). By contrast, all of the rats that received daily injections of BSA exhibited elevated albumin excretion into the urine based on urine samples collected over a 24 h period. Importantly, urine albumin excretion was significantly greater in *Trpc6*^wt/wt^ rats than in *Trpc6*^del/del^ rats (Figure 2). This pattern was observed after either 14 or 28 days of albumin overload. At both time points, two-way ANOVA revealed a robust interaction between the effects of BSA injection and genotype, and this pattern indicated a non-trivial effect of TRPC6 inactivation to reduce albumin excretion in BSA-injected animals. Albuminuria was approximately half as severe in *Trpc6*^del/del^ rats compared to their *Trpc6*^wt/wt^ littermates, indicating that TRPC6 plays a role in regulating glomerular permeability to albumin. However, blood urea nitrogen levels remained within the normal range in all treatment groups and genotypes, and there was no statistically significant difference between any of the treatment groups (Figure 2C). Albumin overload did not affect body weight, but resulted in an increase in kidney weight:body weight ratios in *Trpc6*^del/del^ and *Trpc6*^wt/wt^ rats (Figure 2D). There was at least a trend for this to be less severe in the *Trpc6*^del/del^ rats.

**Figure 3:**
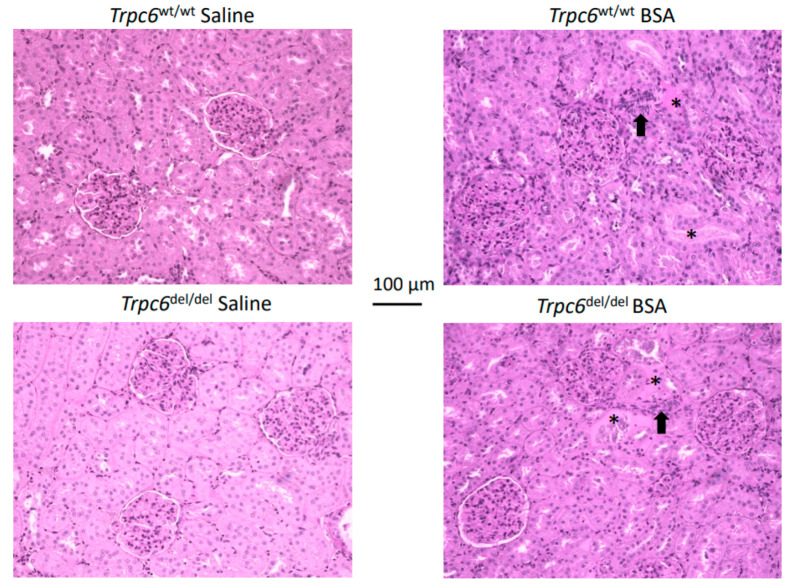
Representative PAS-stained sections from animals in each group. BSA injection did not produce glomerulosclerosis to any extent in either genotype. However, there were many indications of tubulointerstitial disease that could be seen with this staining, especially regions or marked hypercellularity (examples shown by arrows), as well as hyalinization of some tubules (asterisks).

However, the histological changes in PAS-stained sections of the renal cortex were not very severe after 28 days of protein overload, compared to what was observed in the chronic PAN nephrosis [18] or anti-GBM glomerulonephritis models [21]. In particular, we did not observe much glomerulosclerosis in the albumin overload model (Figure 3), which may reflect the fact that these experiments were performed on the Sprague Dawley background. Instead, we observed changes within tubulointerstitial compartments, including marked hypercellularity and occasional hyalinization of tubules (Figure 3).

**Figure 4:**
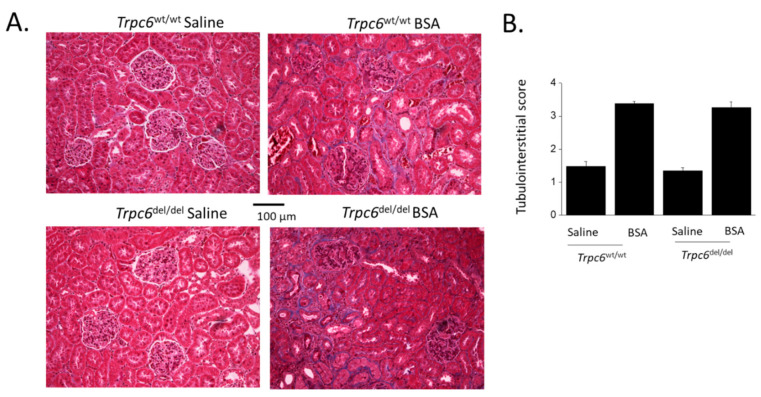
Results from representative sections stained using Masson’s trichrome method from animals in each group after 28 days of treatments. BSA injection produced marked tubulointerstitial fibrosis that can be seen as increased collagen deposition (blue) in tubulointerstitial compartments in the representative micrographs (**A**). Semi-quantitative analysis of these images revealed no protective effect of TRPC6 inactivation (**B**).

**Figure 5:**
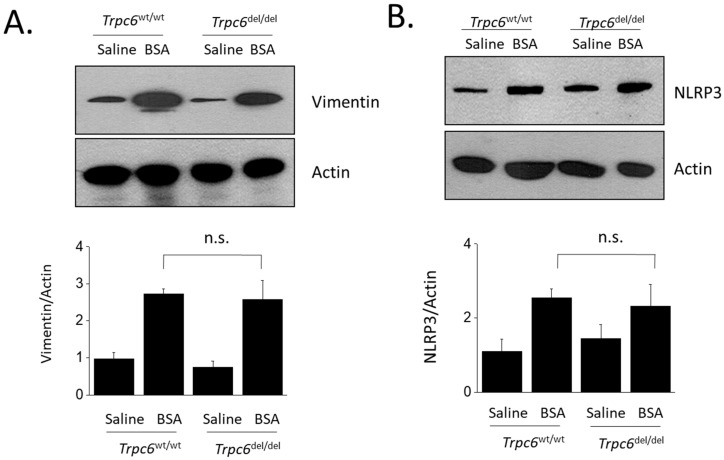
Immunoblot analysis showing expression of tubulointerstitial disease markers in renal cortex after 28 days of treatments. BSA injections resulted in marked increases in vimentin (**A**) and NLRP3 (**B**), and these changes occurred to the same extent in wild-type animals and animals with TRPC6 inactivation. Representative immunoblots are shown above densitometric analyses showing mean ± SD.

TRPC6 inactivation did not appear to produce significant protection of the tubulointerstitial compartment from changes induced by albumin overload. Thus, by evaluating tubulointerstitial fibrosis using Masson’s trichrome stain, we observed fibrosis in animals subjected to albumin overload (Figure 4A). A semi-quantitative analysis of these data revealed a significant effect of BSA injection to increase tubulointerstitial disease, but this was not affected by the genotype of the animal (Figure 4B). A similar pattern was seen when we examined renal cortical expression of biochemical markers of tubulointerstitial disease, such as vimentin (Figure 5A) or NLRP3 (Figure 5B). Sustained protein overload increased all of these disease markers and TRPC6 inactivation did not ameliorate these changes.

**Figure 6:**
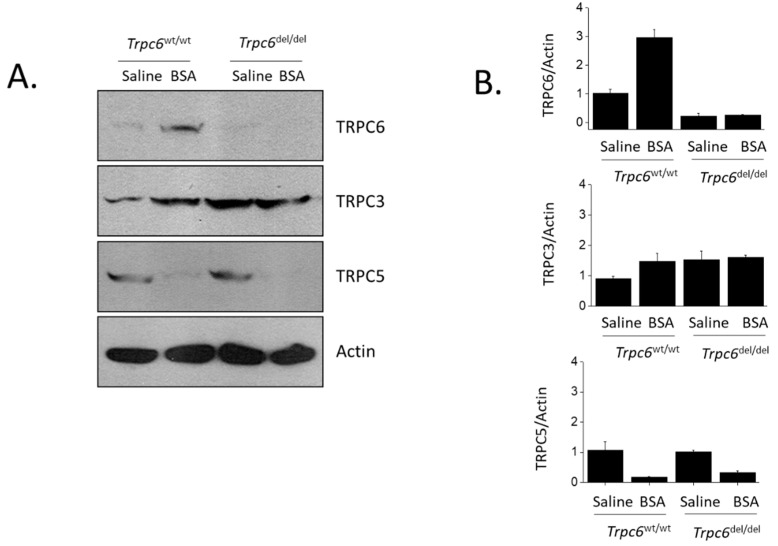
Immunoblot analysis showing abundance of TRPC family channels in renal cortex after 28 days of treatments. In wild-type rats, BSA injections resulted in marked increases in TRPC6 and TRPC3 abundance, but resulted in a near complete loss of TRPC5. Note that TRPC6 was barely detectable in *Trpc6*^del/del^ rats, as previously described in detail [18]. Increases in TRPC3 and a nearly complete loss of TRPC5 were observed in BSA-treated rats of both genotypes. (**A**) Immunoblots from representative animals. (**B**) Results of densitometric analyses indicating mean ± SD for each type of channel as indicated.

In this study, we also examined the effects of albumin overload on the abundance of TRPC family channels in renal cortex by immunoblot analysis. We observed that *Trpc6*^wt/wt^ rats chronically treated with BSA had markedly greater abundance of TRPC6 in renal cortex compared to *Trpc6*^del/del^ littermates (Figure 6A). As reported previously [18], only trace amounts of TRPC6 protein could be detected in *Trpc6*^del/del^ rats, and those channels were non-functional. BSA injection also increased TRPC3 abundance in *Trpc6*^wt/wt^ rats compared to saline-treated controls. As we reported previously [18], TRPC3 abundance is increased in *Trpc6*^del/del^ rats, and we noted that this did not increase further as a result of BSA injection (Figure 6B). Interestingly, and quite surprisingly, BSA treatment caused an almost complete loss of TRPC5 expression in renal cortex as assessed by immunoblot. This occurred to the same extent in both *Trpc6*^wt/wt^ and *Trpc6*^del/del^ rats.

## 4. Discussion

A role for TRPC6 channels in the pathophysiology of acquired glomerular disorders can be inferred when some aspect of their expression or function is dysregulated in a human disease or in an animal disease model, and when inhibition or deletion of TRPC6 channels ameliorates either the disease process or some physiological index of glomerular filtration. In the present study, we obtained three robust observations. First, we observed a marked increase in the abundance of TRPC6 and TRPC3 channels in the renal cortex of wild-type Sprague Dawley rats subjected to protein overload caused by daily injections of BSA. Second, protein overload resulted in a nearly complete loss of TRPC5 channels. Third, we observed that rats that lack functional TRPC6 channels have substantially reduced albuminuria in response to protein overload. However, the reduction in urine albumin excretion was not sufficient in and of itself to produce comparable reductions in histological or biochemical indices of tubulointerstitial disease or azotemia.

The reduction in urine albumin excretion in *Trpc6*^del/del^ rats provides additional evidence that TRPC6 plays some role in regulating the function of the glomerular filtration barrier, which could occur as a result of effects on podocytes, mesangial cells, or both [1,18,21,22]. This effect was comparable in amplitude to effects on urine albumin excretion in several other animal models [1], i.e., it reduces albuminuria by about half. However, this means that urine albumin excretion remains far above normal even in *Trpc6*^del/del^ rats. Glomerulosclerosis as ascertained by light microscopic histology in the albumin overload model is minimal, at least on the Sprague Dawley background, and therefore the experimental design did not allow us to ascertain if TRPC6 inactivation can exert a glomeruloprotective effect similar to those that occur during anti-GBM glomerulonephritis [21], in aging [22], and in chronic PAN nephrosis [18]. In addition, the albumin overload animals did not exhibit significant azotemia in the present study, suggesting that a sufficient number of functional nephrons remained to prevent significant stress on glomeruli in the remaining nephrons.

The albumin overload model has long been known to produce tubulointerstitial fibrosis and inflammation in rats [28,29,30,31], as is seen in the present study, although the extent to which this occurs depends on the genetic background, and is much more severe in Wistar rats than on the Sprague Dawley background used here [29]. The mechanisms that cause this effect are complex and not entirely understood. There is considerable evidence that a portion of the tubulointerstitial disease occurs secondary to increased albumin uptake by tubular cells; in other words, it is the proteinuria itself that ultimately drives tubulointerstitial disease [30]. However, it has been observed by electron microscopy that albumin overload causes the appearance of structures that resemble protein reabsorption droplets within glomerular cells [31]. Here, we noted that, in spite of the reduction in albuminuria, we could not detect any protective effect of TRPC6 inactivation on tubulointerstitial disease based either histology or on biochemical markers that were elevated as a result of albumin overload. One possible explanation is that the reduction in albuminuria caused by TRPC6 inactivation was not sufficient to inhibit the inflammatory and fibrotic processes driven by abnormally high protein reabsorption by tubular cells. This could occur if tubular endocytic processes are saturated even in the face of the reduced albumin concentrations in the glomerular ultrafiltrate of *Trpc6*^del/del^ rats. Another possible explanation is that protein reuptake by glomerular cells plays a role in driving tubulointerstitial disease caused by albumin overload. Thus, it is possible that podocytes and/or mesangial cells respond to markedly increased endocytotic loads by increasing secretion of pro-inflammatory molecules, which then trigger inflammatory and fibrotic processes within the tubulointerstitial compartment. Those effects could persist, even if there is a reduction in albumin entering the ultrafiltrate. In this regard, it has been reported that hospitalized patients with markedly increased serum albumin concentrations have a markedly higher risk for acute kidney injury [41]. Albumin uptake by podocytes is known to trigger an increases in synthesis and secretion of several pro-inflammatory molecules, including cyclooxygenase-2 [42] and TGFβ1 [35], as well as TNF and IL-6 [43].

While significant protective effects in the interstitium were not evident in the present study, is now well established that TRPC6 knockout in mice reduces tubulointerstitial fibrosis evoked by unilateral ureteral obstruction (UUO) [26,27]. It is possible that the anti-fibrotic effect of TRPC6 knockout or inhibition is simply more robust in mice than it is in rats. However, it is also possible that the effect of TRPC6 inactivation depends on the intensity and or location of the stimulus that triggers the disease. UUO produces a severe and immediate injury in cells located throughout the entire nephron, which are simultaneously subjected to substantial mechanical, metabolic, and immunological stresses, leading to interstitial disease within days [44]. UUO may therefore be more likely to engage cells that utilize TRPC6 channels as part of the pro-fibrotic and pro-inflammatory cascades in response to this insult. We previously observed that TRPC6 inactivation reduced tubulointerstitial disease in chronic PAN nephrosis [18]. In that model, the initial effect is a loss of a substantial number of podocytes, and it is likely that the tubulointerstitial disease is triggered by severe inflammatory events occurring close to the boundary of Bowman’s capsule and the proximal tubule. In any case, from the point of view of therapeutic targeting, protective effects in any renal compartment, and reductions in urine albumin excretion, are worth pursuing.

As with other kidney disease models that we examined, protein overload in wild-type rats was associated with a marked increase in the net abundance of TRPC6 and TRPC3 in renal cortex. However, we were surprised to observe that protein overload resulted in an almost complete loss of TRPC5 in renal cortex as assessed by immunoblot. This effect was not ameliorated by TRPC6 inactivation. In the glomerular disease models that we previously examined, we found that TRPC5 abundance neither increased nor decreased relative to what was seen in control animals [18,21,22], and we do not know the mechanisms leading to loss of TRPC5 in protein overload. To date, the role of TRPC5 in the normal function of the kidney and in the pathogenesis of kidney disease is not clear (reviewed in [1]). There are reports that TRPC5 knockout and TRPC5 inhibitors can reduce the severity of albuminuria and glomerulosclerosis in certain kidney disease models in rats and mice [45,46,47], suggesting that excessive TRPC5 function is pathogenic in glomeruli. However, protective effects of pharmacological TRPC5 inhibition have not been seen by all investigators [48], and the over-expression of either wild-type or dominant-negative TRPC5 channels in glomeruli does not appear to induce kidney disease [48], in contrast to what is seen with over-expression of TRPC6 [16,17]. In the albumin overload model studied in the present work, an increase in overall TRPC5 activity is highly unlikely to contribute to the disease process, but it is possible that the loss of TRPC5 is a contributing factor.

In summary, we observed that albumin overload in Sprague Dawley rats causes marked increases in TRPC6 abundance in the renal cortex, accompanied by a nearly complete loss of TRPC5; and that TRPC6 inactivation reduces albuminuria, but does not affect tubulointerstitial fibrosis or inflammation. The limitations of the present study include the fact that TRPC6 inactivation in these animals is constitutive and global, and that the albumin overload model is not a precise analog to any human disease.
